# Decreased Retinal Vascular Density in Alzheimer’s Disease (AD) and Mild Cognitive Impairment (MCI): An Optical Coherence Tomography Angiography (OCTA) Study

**DOI:** 10.3389/fnagi.2020.572484

**Published:** 2021-01-15

**Authors:** Xi Wang, Qianhua Zhao, Rui Tao, Huimeng Lu, Zhenxu Xiao, Li Zheng, Ding Ding, Saineng Ding, Yichen Ma, Zhaozeng Lu, Yiqin Xiao

**Affiliations:** ^1^Department of Ophthalmology, Huashan Hospital, Fudan University, Shanghai, China; ^2^Department of Neurology, Huashan Hospital, Fudan University, Shanghai, China; ^3^National Clinical Research Center for Aging and Medicine, Huashan Hospital, Fudan University, Shanghai, China

**Keywords:** Alzheimer’s disease, mild cognitive impairment, optical coherence tomography angiography, retinal thickness, retinal blood flow

## Abstract

**Background:**

To explore the retinal vascular density changes in Alzheimer’s disease (AD) and mild cognitive impairment (MCI) patients using optical coherence tomography angiography (OCTA).

**Methods:**

We recruit 62 AD patients, 47 MCI patients, and 49 cognitively healthy controls (HC) in this study. All participants in the study received a comprehensive ophthalmological and neurological evaluation, including global cognitive screening, as well as the Mini-Mental State Examination (MMSE), and completed the following eye examinations: visual acuity (VA), intraocular pressure (IOP), examination with slit-lamp, fundus photography (Version 1.5.0.0, NIDEK CO, LTD) and Optical coherence tomography imaging (software ReVue version 2017.1.0.155, Optovue Inc., Fremont, CA, United States). The visual rating scales for atrophy and white matter lesion in MRI was evaluated for all the patients with AD and MCI.

**Results:**

In the AD patient group, the superficial vascular density in the superior, inferior and whole retina was 44.64 ± 3.34, 44.65 ± 3.55, and 44.66 ± 3.36, respectively. These values were 44.24 ± 3.15, 43.72 ± 3.16, and 44 ± 3.07, respectively, in the MCI patient group. After multivariate analysis of the generalized linear model, adjustments for the confounding factors of sex, age, hypertension, diabetes and the quality index of OCTA image, the superficial vascular density in the AD and MCI patient groups was significantly lower than that in the HC group (*P* < 0.05): 46.94 ± 2.04, 46.67 ± 2.26, and 46.82 ± 2.08, respectively. No difference in the area of the FAZ among the three groups was observed (AD group: 0.34 ± 0.11 mm^2^; MCI group: 0.36 ± 0.12 mm^2^; control group: 0.33 ± 0.12 mm^2^, *p* > 0.05). The ganglion cell complex (GCC) thickness, inner parafovea thickness, and peripapillary retinal nerve fiber layer (p-RNFL) thickness were associated with the superficial vascular density. We found no significant correlation between the global cognition (MMSE scores) or between the Fazekas score and retinal OCT angiogram flow density.

**Conclusion:**

The superficial vascular density in the AD and MCI patient groups was significantly lower than that in the HC group. Our findings suggest the retinal microvascular dysfunction occurred in MCI and AD.

## Introduction

Globally, approximately 47 million people suffer from dementia, and the number is expected to triple in 2050 (Livingston et al., 2017). China has the largest population of dementia patients in the world, imposing a heavy burden on the public and health-care system (Jia et al., 2020). Alzheimer’s disease (AD), as the most common type of dementia, is characterized by its continuous, irreversible pathological process, which initiates decades before overt cognitive impairment (Sperling et al., 2011). Mild cognitive impairment (MCI) is the prodromal stage of AD (Morris et al., 2001; Langa and Levine, 2014). Individuals with MCI are at high risk of developing dementia. Although cerebrospinal fluid (CSF) analyses (amyloid and tau proteins) and positron emission tomography (PET) scans may help in the early identification of MCI (Albert et al., 2011; Ottoy et al., 2019), these methods have not been widely used in clinical practice due to their invasiveness and high cost (Handels et al., 2017). Economic, non-invasive, precise early markers are still needed.

Alzheimer’s disease is also linked to many vascular risk factors, such as diabetes, stroke, atherosclerosis, and hypertension (Vos et al., 2015). A cross-sectional study involving 1,143 subjects showed that cerebral atherosclerosis and arteriosclerosis were associated with AD and dementia (Arvanitakis et al., 2016). Recent studies have found an association of cerebral blood flow (CBF) with the pathogenesis of AD, suggesting that it may serve as a biomarker for preclinical AD (Hays et al., 2016). In patients with MCI and AD, cerebral hypoperfusion has been reported to increase cerebral vascular tortuosity and decrease vascular density (Yamashita et al., 2014).

Although there is evidence that vascular injury plays a role in the pathogenesis of AD and MCI, it is unclear whether vascular changes cause neurological death directly or whether they are secondary effects of reduced neuronal metabolism. However, the cerebral blood vessels are too small to be directly observed and assessed *in vivo* (Smith and Beaudin, 2018). Because the retinal and cerebral vasculatures are similar in embryonic development and physiological and anatomical features, researchers have focused on investigating intraocular vascular structures to understand cerebral vasculature (Berisha et al., 2007). Retinal Laser Doppler flowmetry has been used to detect venous stenosis and decreased blood flow in patients with AD and MCI (Feke et al., 2015).

Optical coherence tomography angiography (OCTA) is a new angiographic technology based on OCT technology that can visualize and quantify retinal and choroidal vessels without intravenous injection of contrast agent. It can provide high-resolution images of retinal and choroidal microvasculature to visualize the changes in retinal blood vessels. OCTA has more advantages than fundus fluorescein angiography (FFA) and Retinal Laser Doppler flowmetry. It is non-invasive, safer, and faster. It can better display the superficial and deep capillary layers of the retina and can quantitatively measure the non-perfused area of the macula.

Optical coherence tomography angiography capillary perfusion density map and mean perfusion density provide a simple method for grading the gradual changes in blood vessels. During the last 2 years, OCTA has been used in many ocular and systemic diseases. Bulut et al. (2018) were the first to use OCTA to assess retinal vascular changes in AD. They found that vessel density was significantly reduced in all regions of the AD group (*P* < 0.05), and this reduction was correlated with Mini-Mental State Examination (MMSE) scores (Bulut et al., 2018). Many studies have found that vessel density decreases in the deep vascular plexus (DVP) and superficial vascular plexus (SVP) in AD patients (Jiang et al., 2018; Zabel et al., 2019b). In particular, the reduced blood flow density of the retina was related to the cerebrovascular disease of AD but not to the pathology of AD (Lahme et al., 2018). However, there are also different findings. van de Kreeke et al. (2020) found that Aβ+ individuals had significantly higher vessel density than Aβ− individuals in all regions. At present, only a few studies pay attention to the changes in retinal blood flow in MCI. Jiang et al. (2018) found that the density of retinal microvessels of the DVP in the superior nasal quadrant was significantly lower in patients with MCI than in the control group.

This study aimed to evaluate retinal macular area and optic nerve head (ONH) perfusion in patients with MCI and early AD using OCTA. In addition, we quantified the association of OCTA findings with retinal thickness. A direct comparison of OCTA analysis in patients with AD and MCI could make a significant contribution to understanding the pathophysiology of these neurodegenerative diseases and may help to elucidate the underlying cause of the thinning of the peripapillary retinal nerve fiber layer (p-RNFL). We hypothesized that retinal vascular pathology contributes to downstream neurodegeneration. We wanted to explore whether retinal blood flow changes in MCI and early AD patients and, if so, whether this change correlates with changes in retinal nerve thickness and with cognitive scores and cerebrovascular lesions.

## Materials and Methods

### Participants

A total of 47 MCI patients, 49 age-matched healthy controls (HC), and 62 AD patients participated in this study. The patients were enrolled from the memory clinic in Huashan Hospital from December 2017 to March 2018. HC subjects were recruited from the Shanghai Aging Study, a community-based aging cohort. AD and MCI were diagnosed based on the clinical evaluation, neuropsychological assessment, neuroimaging (such as cranial MR or CT), and laboratory tests according to the 2011 guidelines of the National Institute of Aging-Alzheimer’s Association workgroups (NIA/AA). The HC subjects had no evidence of neurological disorders and were determined to be cognitively healthy according to neuropsychological assessment. The interval between the ophthalmic examination and clinical evaluation was 1–90 days. The study was approved by the Huashan Hospital Institutional Review Board (HIRB). All participants were informed of the study protocol in detail and signed the informed consent form. All participants were treated under the tenets of the Declaration of Helsinki.

All participants in the study underwent global cognitive screening, as well as the MMSE, and completed the following eye examinations: visual acuity (VA), intraocular pressure (IOP), examination with slit-lamp, fundus photography (Version 1.5.0.0, Nidek Co, Ltd) and OCTA. Patients with MCI or AD had to undergo a comprehensive battery of tests that covered four main domains: (1) memory, (2) executive function/attention, (3) language, and (4) visuospatial skills. Normative data and detailed descriptions of these tests are reported elsewhere (Karakosta et al., 2012; Xu et al., 2018). MCI and AD were diagnosed based on the 2011 NIA/AA guidelines. The HC subjects had no evidence of a neurological disorder or cognitive complaints.

For all groups, the exclusion criteria were as follows: (1) personal or family history of psychiatric disorders, Lewy body dementia, vascular dementia, frontal temporal dementia, multiple sclerosis, or Parkinson’s disease,; (2) a history of glaucoma, retinal detachment, optic neuropathy, other optic nerve diseases and retinal vascular disorders such as age-related macular degeneration, ocular trauma, a cataract that disturbs visual and OCT examination, or other ocular disorders; (3) a history of carbon monoxide poisoning, alcohol abuse, hypothyroidism, or other serious chronic medical conditions; and (4) severe cognitive impairment rendering the individual unable to cooperate in the eye examination.

### Optical Coherence Tomography Imaging

**FIGURE 1:**
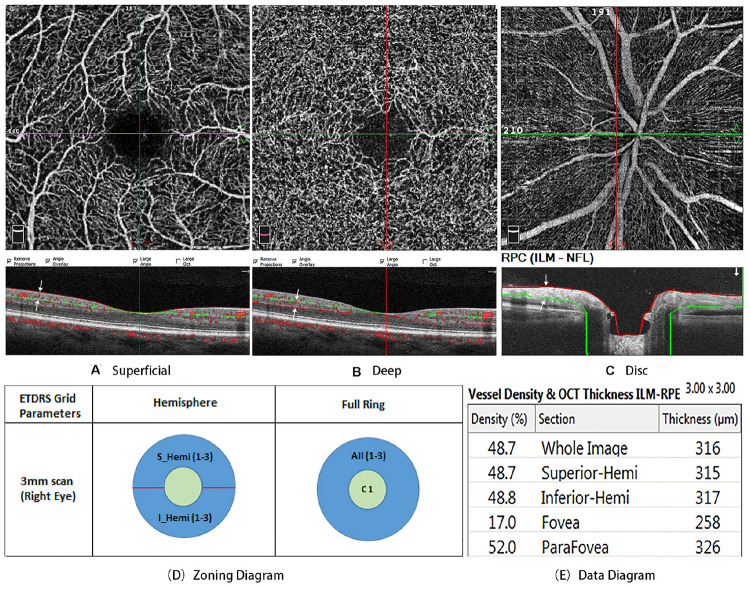
**(A)** is the superficial en face image, which was segmented with an inner boundary at the internal limiting membrane (red line) and an outer boundary set at 10 mm above the inner plexiform layer (green line). **(B)** is the deep en face image, which segmented with an inner boundary at 10 mm above the inner plexiform layer (green line) and an outer boundary at 10 mm beneath the outer plexiform layer (red line). **(C)** is the radial peripapillary capillaries (RPCs), which was defined as extending from the internal limiting membrane to the posterior boundary of the RNFL. **(D)** is Zoning diagram two different modes of data analysis: hemisphere and full ring. **(E)** is data diagram. All scans were reviewed independently by two investigators to ensure correct segmentation and sufficient image quality.

Optical coherence tomography angiography imaging of all subjects was performed with the Optovue Angiovue System (software ReVue version 2017.1.0.155, Optovue Inc., Fremont, CA, United States). The device obtains volumetric scans of 304 × 304 A-scans at 70,000 A-scans per second in approximately 3.0 s. Two B-scans were captured at each fixed position before proceeding to the next sampling location, and two orthogonal OCTA volume scans were used to minimize motion artifacts and fixation changes. Macula imaging was performed using a 3 × 3 mm^2^ scan and images of the ONH, and the peripapillary area was obtained with a 4.5 × 4.5 mm^2^ scan. The software automatically segmented and calculated these full-thickness retinal scans into the superficial and deep inner retinal vascular plexuses, outer retina, and choriocapillaris. Each 3 × 3-mm parafoveal scan area was automatically segmented as follows: The superficial en face image was segmented with an inner boundary at the internal limiting membrane and an outer boundary set at 10 mm above the inner plexiform layer, whereas the deep en face image was segmented with an inner boundary at 10 mm above the inner plexiform layer and an outer boundary at 10 mm beneath the outer plexiform layer. All scans were reviewed independently by two investigators (XW and RT) to ensure correct segmentation and sufficient imaging quality (Figures 1A,B). The foveal avascular zone (FAZ) was also automatically identified.

Macular vessel density was analyzed throughout the area of 3 × 3 mm^2^. Two different modes of data analysis were used: (1) the software automatically divided the macular retina into superior and inferior regions and (2) two circular areas from the center to the outside were calculated, the fovea (Ø = 1 mm) and the parafovea (Ø = 3 mm) (Figures 1D,E). The density of the radial peripapillary capillaries (RPCs) was defined as extending from the internal limiting membrane to the posterior boundary of the RNFL (Figure 1C). We used the Optovue AngioVue System to investigate retinal thickness by measuring the following indexes: p-RNFL thickness and ganglion cell complex (GCC) thickness.

All the measurements were completed by one ophthalmologist. Images were independently reviewed by two ophthalmologists, and only those with good quality were included (image quality > 6). Measurements with motion artifacts present on the en face images were also rejected. For each participant, both eyes were examined. However, to ensure the independence of variables, calculated means of eye results were included in the statistical analysis. The right or left eye was chosen if the other eye was ineligible. The ophthalmologists were blinded to the diagnosis in both the image review phase and the data analysis phase.

### Neuroimaging Data Acquisition and Imaging Analysis

To ensure the uniformity of data collection, we performed brain MR imaging through standard procedures. A brain scan was conducted in a Siemens 3.0 T MAGNETOM Verio with an MPRAGE T1WI sequence. The voxel size was 1 × 1 × 1 mm. The FreeSurfer^1^ Analysis software package was used to analyze and visualize structural neuroimaging data obtained from cross-sectional or longitudinal views. The procedures used for MRI structural analysis were as follows: (1) OsiriX^2^ software was used to view the obtained brain MRI structure and exclude data produced by apparent lesions and imaging artifacts; (2) FreeSurfer software was used to analyze the brain MRI structure screened by recon-all instructions; (3) tKmedit and tKsurfer were used to check the partition of white-gray matter after checking recon-all.log to exclude any errors; and (4) Asegstats2table and aparcstats2table were used to extract data for the relevant structure.

The examination timepoints of OCTA and MRI scans were within a six-month period. The Fazekas scale was independently used by two experienced observers who were blinded to clinical information. A 0–3 rating scale was used to quantify the amount of white matter T2 hyperintense lesions, which are often attributed to chronic small vessel ischemia (Fazekas et al., 1987; Scheltens et al., 1998).

### Statistics

IBM SPSS Statistics software (vision 24) was used in the data analysis. Continuous parameters are expressed as the mean and the standard deviation (SD), and categorical parameters are shown as number and frequencies (%). Data were tested for normal distribution by histograms and Q–Q plots. Normally distributed data were analyzed with one-way ANOVA, non-normally distributed data were analyzed with a Mann–Whitney *U* test, and binary variables were analyzed with Fisher’s exact test. For multiple comparisons, Bonferroni correction was used if necessary. The *post hoc* test was adopted for the ANOVA, and the Dwass, Steel, Critchlow-Fligner multiple comparison procedure (DSCF) was used for the Kruskal–Wallis test. Linear regression was used to assess the association between OCTA and retinal thickness measures. The generalized linear model (GLM) was used to evaluate the association between the study groups and the OCTA measures to adjust for age, sex, hypertension, diabetes and the quality index of OCTA image. Two-sided *p*-values and 95% CIs were used in SPSS software. Significance was determined at *p* < 0.05.

## Results

### Characteristics

A total of 191 subjects were referred for the study. After meeting the inclusion criteria, retinal blood flow and thickness measurements were successfully obtained in 165 subjects. Of the remaining subjects, seven of these individuals did not achieve OCTA image quality >6. Data from these subjects were excluded from the analysis. As a result, data from 158 subjects were used in the analysis.

The demographic and clinical characteristics of subjects in the AD, MCI, and HC groups are shown in Table 1. All groups were comparable in age, sex, VA, IOP, HBP, DM ratio, and visual rating scales (MTA, Fazekas, GCA and PCA) (*p* > 0.05). Compared with the MCI and HC groups, the AD cohort had a significantly worse MMSE score (*p* < 0.01).

**TABLE 1:** Demographic and clinical characteristics of the study subjects.

	Patients with AD	Patients with MCI	Control subjects	*p*-value
			
	(*n* = 62)	(*n* = 47)	(*n* = 49)	
Age	71.81 ± 7.98	72.73 ± 7.75	69.50 ± 5.94	0.079
Medium (range)	73 (57, 89)	74 (53, 87)	71 (57, 81)	
Sex (M/F)	27/35	18/29	17/32	0.634
VA	0.68 ± 0.25	0.75 ± 0.22	0.70 ± 0.14	0.275
IOP (mmHg)	14.24 ± 2.55	14.62 ± 2.57	14.26 ± 2.20	0.718
HBP	22	19	18	0.865
DM	8	8	8	0.812
MMSE score	19.92 ± 4.54	28.04 ± 1.90	28.67 ± 1.0	<0.001**
Medium (range)	20 (7, 24)	28 (23, 30)	29 (27, 30)	
CDR global	1.339 ± 0.061	0.532 ± 0.018	0.031 ± 0.017	<0.001**
MTA	1.77 ± 0.231	1.34 ± 0.143	0.89 ± 0.200	0.071
Fazekas	0.88 ± 0.234	0.71 ± 0.137	0.56 ± 0.176	0.598
GCA	1.46 ± 0.144	1.14 ± 0.147	0.780 ± 0.147	0.108
PCA	1.08 ± 0.178	0.76 ± 0.137	0.44 ± 0.176	0.257

Data reported as mean and SD (standard deviation) with *p* values. AD, Alzheimer’s disease; MCI, mild cognitive impairment; M, male; F, female; VA, visual acuity; IOP, intraocular pressure; HBP, high blood pressure; DM, diabetes mellitus; MMSE, Mini–Mental State Examination; CDR, clinical dementia rating, the global score of CDR was used. The bold values indicate statistical significance. **p < 0.01.

### OCTA Findings

The macular flow densities determined by retinal OCT angiogram are summarized in Table 2. In the AD patient group, the superficial vascular density in the superior, inferior and whole retina was 44.64 ± 3.34, 44.65 ± 3.55, and 44.66 ± 3.36, respectively. These values were 44.24 ± 3.15, 43.72 ± 3.16, and 44 ± 3.07, respectively, in the MCI patient group. The superficial vascular density in the AD and MCI patient groups was significantly lower than that in the HC group (*P* < 0.05): 46.94 ± 2.04, 46.67 ± 2.26, and 46.82 ± 2.08, respectively. In the AD patient group, the deep vascular density in the superior, inferior and whole retina was 49.53 ± 3.48, 49.36 ± 3.51, and 49.42 ± 3.4, respectively, while these values were 49.69 ± 2.84, 49.46 ± 3.05, and 49.57 ± 2.89, respectively in the MCI patient group. The deep vascular density in the AD and MCI patient groups was significantly lower than that in the HC group (*P* < 0.05): 50.98 ± 2.89, 50.78 ± 2.89, and 50.89 ± 2.86, respectively. No difference in the area of the FAZ among the three groups was observed (AD group: 0.34 ± 0.11 mm^2^; MCI group: 0.36 ± 0.12 mm^2^; control group: 0.33 ± 0.12 mm^2^, *p* > 0.05).

**TABLE 2:** Comparison of vascular density in macular among the three groups.

	Patients with AD	Patients with MCI	Healthy control subjects	*p*-value	
				
	Mean ± SD	Mean ± SD	Mean ± SD	AD vs HC	MCI vs HC
**OCT-A deep (%)**					
Whole enface	49.423.4	49.572.89	50.892.86	0.016*	0.041*
Superior	49.533.48	49.692.84	50.982.89	0.018*	0.049*
Inferior	49.363.51	49.463.05	50.782.89	0.023*	0.046*
Fovea	28.536.8	26.837.11	28.946.7	0.758	0.137
Parafovea	52.023.65	52.362.96	53.42.77	0.027*	0.118
**OCT-A superficial (%)**					
Whole enface	44.663.36	443.07	46.822.08	<0.001**	<0.001**
Superior	44.643.34	44.243.15	46.942.04	<0.001**	<0.001**
Inferior	44.653.55	43.723.16	46.672.26	0.001**	<0.001**
Fovea	15.895.34	14.095.21	16.185.27	0.776	0.057
Parafovea	47.73.76	47.123.35	49.862.26	0.001**	<0.001**
FAZ (mm^2^)	0.340.11	0.360.12	0.330.12	0.937	0.271

Data reported as mean and SD (standard deviation) with *p* values. The bold values indicate statistical significance. *p < 0.05 and **p < 0.01.

**FIGURE 2:**
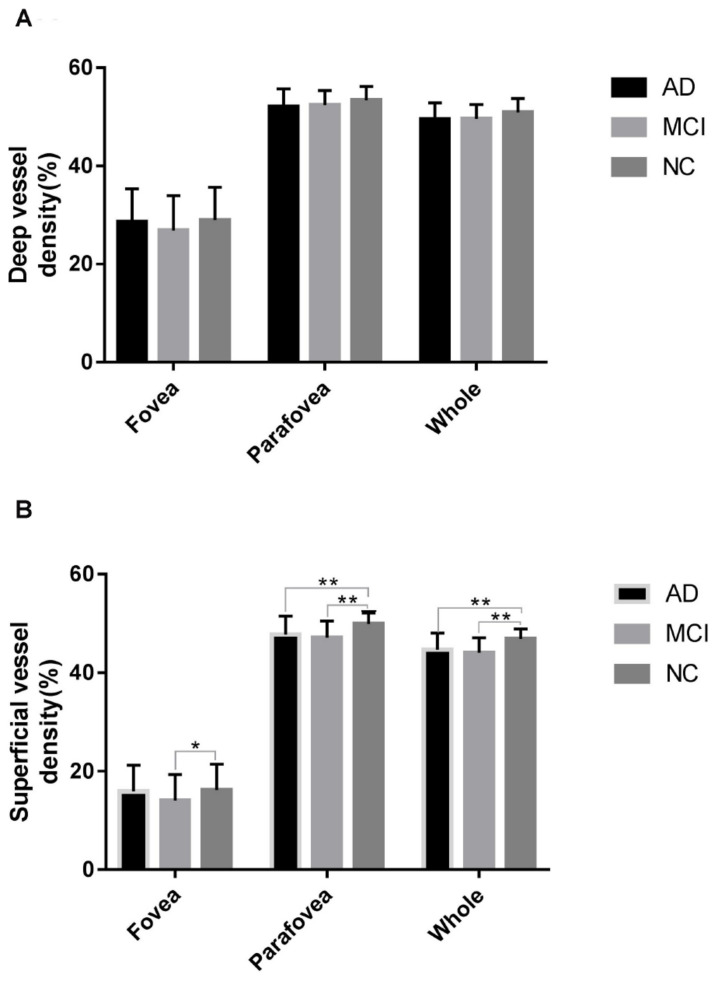
Comparison of vascular density in the fovea, parafovea, and whole areas in the studied groups within the deep retinal vascular plexuses **(A)** and superficial retinal vascular plexuses **(B)**. *p < 0.05 and **p < 0.01.

However, after multivariate analysis by a generalized linear model and after adjustments of the confounding factors of sex, age, hypertension, diabetes and the quality index of OCTA image, as shown in Table 3 and Figure 2, only the superficial vascular density in the AD and MCI patient groups was significantly lower than that in the HC group. The analysis of vessel density in the RPC layer on the whole en face image and the peripapillary area did not differ significantly among the three groups (AD group: 48.38 ± 2.19; MCI group: 48.65 ± 1.92; HC group: 48.33 ± 6.99, *p* > 0.05) in Tables 4, 5.

**TABLE 3:** Multivariate analysis of retinal vascular density.

	AD versus HC			MCI versus HC		
		
	β	95% CI	p-value	β	95% CI	p-value
**OCT-A deep**						
Whole enface	–0.2616	(−0.8053, 0.2821)	0.3456	–0.7413	(−1.7874, 0.3049)	0.1649
Superior	–0.2539	(−0.8085, 0.3007)	0.3695	–0.7088	(−1.7471, 0.3294)	0.1809
Inferior	–0.2365	(−0.7966, 0.3237)	0.4080	–0.7479	(−1.8377, 0.3418)	0.1786
Fovea	–0.0276	(−1.3514, 1.2962)	0.9674	–2.3619	(−5.1648, 0.4410)	0.0986
Parafovea	–0.2793	(−0.8427, 0.2840)	0.3311	–0.5114	(−1.5594, 0.5365)	0.3388
**OCT-A superficial**						
Whole enface	–0.7020	(−1.2254, −0.1787)	0.0086**	–2.0889	(−2.9873, −1.1906)	<0.0001**
Superior	–0.7614	(−1.2756, −0.2472)	0.0037**	–1.9547	(−2.8576, −1.0518)	<0.0001**
Inferior	–0.6394	(−1.2015, −0.0774)	0.0258*	–2.2304	(−3.1875, −1.2734)	<0.0001**
Fovea	0.0082	(−1.0325, 1.0488)	0.9877	–2.1499	(−4.2692, −0.0306)	0.0468*
Parafovea	–0.6451	(−1.2228, −0.0673)	0.0286*	–1.9629	(−2.9529, −0.9730)	0.0001**
**OCT-A disk**						
Whole enface	0.1561	(−1.1079, 1.4202)	0.8087	0.6946	(−1.4836, 2.8728)	0.5320
Peripapillary	–0.3611	(−0.9918, 0.2697)	0.2619	–0.5785	(−1.7105, 0.5534)	0.3165

A generalized linear model was used to evaluate the relationship between retinal measures and clinical diagnosis, which was adjusted for confounders of sex, age, medical history of hypertension and diabetes, and the quality index of OCTA image. The scale parameter was estimated by maximum likelihood. The bold values indicate statistical significance. *p < 0.05 and **p < 0.01.

**TABLE 4:** Comparison of vascular density in ONH among the three groups.

	Patients with AD	Patients with MCI	Healthy control subjects	*p*-value	
				
	Mean ± SD	Mean ± SD	Mean ± SD	AD vs HC	MCI vs HC
**OCT-A disk (%)**					
Whole enface	48.38 ± 2.19	48.65 ± 1.92	48.33 ± 6.99	0.972	0.754
Inside disk	51.48 ± 3.5	50.66 ± 5.14	49.72 ± 4.6	0.106	0.344
Peripapillary	50.71 ± 2.39	50.91 ± 2.33	51.66 ± 2.94	0.123	0.187

*Data reported as mean and SD (standard deviation) with *p* values.*

**TABLE 5:** Multivariate analysis of ONH vascular density.

	AD versus HC			MCI versus HC		
		
	β	95% CI	*p*-value	β	95% CI	*p*-value
**OCT-A disk (%)**						
Inside disk	1.8052	(−2.0813, 0.414)	0.0647	1.3238	(−0.7132, 3.3607)	0.2027
Whole enface	−0.1402	(−2.6865, 2.4061)	0.9141	0.5569	(−1.6346, 2.7484)	0.6184
Peripapillary	−0.8336	(−2.0813, 0.414)	0.1903	−0.6387	(−1.7622, 0.4848)	0.2652

*A generalized linear model was used to evaluate the relationship between retinal measures and clinical diagnosis, which was adjusted for confounders of sex, age, medical history of hypertension and diabetes and the quality index of OCTA image. The scale parameter was estimated by maximum likelihood.*

### Association Analysis

The association between OCTA vascular density measures and retinal thickness is shown in Table 6. The variable was estimated by a linear regression model. We found a significantly positive association between vascular density measures and retinal thickness (*p* < 0.001). Moreover, we also observed a meaningful association between the inner parafoveal thickness and whole and parafoveal superficial vascular density (*p* < 0.001) (Table 6). Regarding the area of the ONH, there was a positive correlation between p-RNFL thickness and the parafovea superficial density (*p* < 0.001). The associations were adjusted for the confounders of sex, age, medical history of hypertension, diabetes and the quality index of OCTA image. The data are shown in Table 6.

**TABLE 6:** Association between the retinal flow density and thickness.

	Whole deep		Parafovea deep		Whole superficial		Parafovea superficial	
				
	β	*p*-value	β	*p*-value	β	*p*-value	β	*p*-value
GCC thickness	–0.053	0.165	–0.046	0.235	0.166	<0.001**	0.164	< 0.001**
Full fovea thickness	0.015	0.267	–0.002	0.862	0.016	0.216	0.007	0.607
Inner fovea thickness	0.027	0.322	–0.021	0.454	0.046	0.074	0.023	0.414
Outer fovea thickness	0.021	0.354	0.007	0.763	0.010	0.621	0.003	0.881
Full parafovea thickness	0.006	0.728	0.003	0.843	0.025	0.099	0.028	0.091
Inner parafovea thickness	0.002	0.942	–0.011	0.700	0.088	0.001**	0.093	0.001**
Outer parafovea thickness	0.018	0.520	0.024	0.407	–0.010	0.705	–0.007	0.801
p-RNFL thickness	–0.013	0.606	–0.005	0.852	0.074	0.003**	0.070	0.009**

Linear regression was used to assess the association between OCT-A and retinal thickness measures, adjusted for confounders of sex, age, medical history of hypertension, diabetes and the quality index of OCTA image. RNFL, retinal nerve fiber layer; GCC, ganglion cell complex. The GCC refers to the complex of the macular fovea, 7 mm from the ILM to the IPL. The inner retina is from ILM to IPL and the outer retina is from INL to RPE. The bold values indicate statistical significance. **p < 0.01.

The current study focused on patients with AD and MCI with few vascular burdens, among which the mean score of the Fazekas scale was 0.64 ± 0.727. We found no significant correlation between global cognition (MMSE scores) or between the Fazekas score and retinal OCT angiogram flow density (Table 7).

**TABLE 7:** Correlation between the MMSE, Fazekas scale, and OCTA parameters.

	Deep whole	Deep parafovea	Superficial whole	Superficial
	enface		enface	parafovea
**MMSE**				
*r*	0.03	0.057	–0.072	–0.066
*p*-value	0.744	0.537	0.441	0.474
**Fazekas scale**				
r	–0.073	–0.149	0.04	–0.007
*p*-value	0.644	0.374	0.804	0.962

*The correlation between cognition and OCTA parameters was evaluated using Pearson’s correlation. MMSE, Mini-Mental State Examination; Fazekas scale, a 0–3 rating scale to quantify the amount of white matter T2 hyperintense lesions.*

## Discussion

Cerebral small blood vessel disease is a risk factor for stroke and dementia (Rensma et al., 2018; van der Flier et al., 2018). More importantly, reduced CBF in MCI patients, compared with controls, has been reported to predict the progression to AD in longitudinal studies (Hirao et al., 2005; Hansson et al., 2009; Park et al., 2012). Moreover, the degree of the decrease in parietal rCBF, but not CSF biomarkers, was related to the more rapid development of AD (Hansson et al., 2009).

The retina is regarded as a part of the central nervous system. It is a direct extension of the brain during embryonic development. Our study shows that the superficial retinal blood flow density of the macula under OCT angiography in the AD and MCI groups was significantly lower than that in the control group after adjustment for vascular factors such as diabetes and hypertension. Many other studies of the individual retinal vessel plexuses by OCTA showed that all regions of the SVP and DVP in AD patients had significantly lower retinal vascular density than healthy controls (Bulut et al., 2018; Jiang et al., 2018). However, Haan et al. (2019) found no differences in vessel density between amyloid-positive AD cases and control subjects by OCTA measurements. van de Kreeke et al. (2020) found that retinal vessel density was higher in individuals with preclinical AD. The inconsistent findings might be attributed to the variability of inclusion and exclusion criteria and different sample sizes, cognitive function tests, controls, and confounding factors.

Multiple studies have demonstrated that the retinal thickness of the GCC (Cheung et al., 2015; den Haan et al., 2018) and p-RNFL (Kesler et al., 2011; Marziani et al., 2013; Pillai et al., 2016; Trebbastoni et al., 2016) is decreased in both AD and MCI patients. However, den Haan et al. (2018) found no retinal thinning in amyloid + AD patients. Further studies found that retinal thickness cannot discriminate between healthy individuals and preclinical AD individuals (den Haan et al., 2019a,b). In a recent study involving 930 participants (414 cognitively healthy people, 192 with probable amnestic MCI, and 324 probable AD patients), the results showed that there were no significant differences in retinal thickness in different layers (Sánchez et al., 2020). In one of our earlier articles, we found that the inner retinal layers of the macular area in MCI patients showed significant thinning compared with those in HCs (Tao et al., 2019). Furthermore, in this study, we found a significantly positive correlation between GCC thickness, p-RNFL thickness, and whole superficial vascular density; nevertheless, there was no significant correlation between global cognition (MMSE scores) or between the Fazekas score and retinal OCT angiogram flow density. Therefore, we hypothesized that the decrease in blood flow density might be the subsequent changes in blood flow caused by the atrophy of retinal ganglion cells and the reduction of oxygen consumption. Zabel et al. (2019b) studied the impairment of microvasculature in AD and primary open-angle glaucoma (POAG). They found that there were abnormalities throughout the entire retinal vascular system in both diseases; significant microcirculatory impairment in POAG patients affects superficial vessels. These findings further suggest that changes in retinal vasculature might be present in the prodromal stage of AD, namely, MCI (Zabel et al., 2019b). As the current studies have a cross-sectional design, the causation between disease and blood flow cannot be determined. Querques et al. (2019) demonstrated that AD and MCI patients are characterized by significant impairment of retinal neurovascular coupling, and this impairment is inversely related to the level of amyloid β in the CSF, but they did not find differences in OCTA. Another reason might be the deposition of amyloid β peptide (Aβ) in the retinal blood vessel wall, which could impair the vasculature of both the brain and the retina.

Some researchers found that both clinical and preclinical AD patients showed an expanded FAZ area (O’Bryhim et al., 2018; Zabel et al., 2019b). However, some research showed that there was no change in the FAZ area (Lahme et al., 2018). No difference in the FAZ areas was observed among the three groups in our study. It is known that the FAZ varies significantly among individuals. Even in healthy individuals, FAZ parameters have high variability (Curcio, 2018; Linderman et al., 2018). Factors such as age, race, sex, and axial length can affect the size of the FAZ region (Wagner-Schuman et al., 2011; Sampson et al., 2017). Therefore, the FAZ may not be a sensitive measure. In the same way, it has been pointed out that direct comparison of individual FAZ areas is not very sensitive, and it may be more reliable to observe the individual change in the FAZ over time (Mammo et al., 2015). Considering the differences and changes in the FAZ in individuals, we will follow up with patients in a future study to obtain more robust data.

The current study has some limitations. First, although we established the diagnosis based on a standard clinical procedure, PET or CSF biomarkers were not included, and this could limit the precision level of our diagnosis. Additionally, because of the lack of detection of markers related to amyloid and tau proteins, we cannot analyze the correlation between them and OCT measurements. Second, although there was no significant difference in age among the three groups and the influence of age was corrected through multivariate analysis, the healthy control group was younger than the MCI and AD groups. In future studies, we still need to include older healthy subjects to control for the possible influence of age. In the meantime, both POAG open-angle glaucoma and MCI are chronic, age-related neurodegenerative changes. Glaucoma is one of the risk factors for AD (Chen et al., 2018; Lee et al., 2019; Xu et al., 2019). Zabel et al.’s (2019a) study found that the nerve fiber layer thickness in glaucoma patients without visual field change was similar to that in AD patients. Therefore, although we excluded individuals with high IOP and angle-closure glaucoma in our study, we cannot wholly exclude normal intraocular tension glaucoma, which also requires long-term follow-up. In addition, at present, there are no standardized protocols for OCTA image acquisition; the lack of defined protocols may produce inconsistencies in clinical practice. Some scholars have suggested that the quality of the image might be reduced if the same number of B-scans were used in a widened field of scanning (de Carlo et al., 2015). Therefore, B-scans covering an area of 3 × 3 mm^2^, repeated horizontally and vertically, were used in our study to analyze the macula area. In a previous article (Haan et al., 2019), a correlation between vascular density and quality factor was observed in OCTA scans. A lower quality factor was associated with lower vascular density. However, in this study, we controlled for image quality by using only those with an OCTA image quality above 6. The effect of image quality on the results needs to be considered carefully. On the other hand, our data show that the differences in macular vascular density among controls, MCI and AD patients are not as distinct. Therefore, the use of vascular density currently might not be sufficiently reliable. Finally, due to the study design, we cannot eliminate the possibility of retinal changes over time.

In summary, the current study demonstrates that patients with AD and MCI have a reduced superficial retinal flow density under OCT angiography, suggesting that retinal microvascular dysfunction occurs in MCI and AD. Future studies will be focused on longitudinal follow-up with larger sample sizes.

## Data Availability Statement

The original contributions presented in the study are included in the article/supplementary material, further inquiries can be directed to the corresponding author/s.

## Ethics Statement

The studies involving human participants were reviewed and approved by Huashan Hospital Institutional Review Board (HIRB), Fudan University (Shanghai). The patients/participants provided their written informed consent to participate in this study.

## Author Contributions

XW, QZ, ZL, and YX contributed to the conception and design of the study. XW, RT, and YM organized the database. XW and QZ performed the statistical analysis. HL, ZX, LZ, DD, and SD contributed to AD and MCI assessment and recruitment. All authors contributed to manuscript revision, read, and approved the submitted version.

## Conflict of Interest

The authors declare that the research was conducted in the absence of any commercial or financial relationships that could be construed as a potential conflict of interest.

## Footnotes

1 http://surfer.nmr.mgh.harvard.edu

2 http://www.osirix-viewer.com
